# Circulating cytokines as potential dynamic biomarkers of PD-1 blockade response and prognosis in non-small cell lung cancer

**DOI:** 10.3389/fimmu.2026.1861545

**Published:** 2026-09-07

**Authors:** Bo Peng, Yongyuan Li, Shuyuan Li, Jiajun Pan, Weimin Qian, Qingpeng Li

**Affiliations:** 1 Department of Thoracic Surgery, Xuzhou Central Hospital, Xuzhou Clinical School of Xuzhou Medical University, Xuzhou, Jiangsu, China; 2 Department of Thoracic Surgery, The Affiliated Hospital of Xuzhou Medical University, Jiangsu, China; 3 Department of Thoracic Surgery, Xuzhou First People's Hospital, Jiangsu, China

**Keywords:** immune response, non-small cell lung cancer, PD-1 blockade, predictive marker, prognosis, serum cytokines

## Abstract

Programmed death 1 (PD-1) inhibitors have transformed the treatment of advanced non-small cell lung cancer (NSCLC), but durable responses remain limited to a subset of patients. Conventional biomarkers, including PD-L1 expression and tumor mutational burden, are constrained by tissue accessibility, intratumoral heterogeneity, and limited capacity for dynamic monitoring. Circulating cytokines offer a minimally invasive alternative because they reflect systemic inflammation, antitumor immunity, and treatment-related immune activation. Evidence indicates that serum levels and on-treatment changes in TNF-α, IFN-γ, IL-6, IL-8, IL-17A, IL-1β, IL-2, IL-5, and IL-10 are associated with response, survival, and immune-related adverse events during PD-1 blockade. In general, declines in IL-6, IL-8, IL-17A, and IL-10, together with transient increases in IFN-γ and TNF-α, may indicate favorable immune reinvigoration, although findings remain inconsistent across studies. This review summarizes the biological functions, mechanistic roles, and clinical relevance of circulating cytokines as dynamic biomarkers, evaluates their potential values and current limitations in predicting the efficacy of PD-1 inhibitor therapy in NSCLC.

## 1 Introduction

Non-small cell lung cancer (NSCLC) remains the most prevalent form of lung cancer worldwide (1). The introduction of programmed death 1 (PD-1) inhibitors has transformed the therapeutic landscape of advanced NSCLC by restoring exhausted T-cell function through blockade of the PD-1/PD-L1 immune checkpoint, resulting in durable clinical responses in a subset of patients (2–5). Currently, PD-1 inhibitor-based chemoimmunotherapy is the standard first-line treatment for patients with advanced driver-negative NSCLC, while PD-1 inhibitor monotherapy is recommended for selected patients with PD-L1-positive tumors (6, 7). For patients with a tumor PD-L1 proportion score of ≥1%, PD-1 inhibitor monotherapy is also a feasible treatment option, with greater benefit observed in those with high PD-L1 expression (8). However, treatment responses remain highly heterogeneous. Although biomarkers such as PD-L1 expression, tumor mutational burden, tumor-infiltrating lymphocytes, microsatellite instability, and other molecular features have been investigated, their clinical utility is limited by tissue accessibility, intratumoral heterogeneity, high cost, and the inability to provide dynamic immune monitoring (9, 10).

Circulating cytokines have emerged as promising non-invasive biomarkers because they reflect both systemic inflammatory status and antitumor immune activity (11, 12). Cytokines, including interleukins (ILs), interferons (IFNs), and tumor necrosis factors (TNFs), regulate immune-cell activation, differentiation, and communication through autocrine, paracrine, and endocrine signaling (13, 14), while also influencing tumor proliferation, angiogenesis, metastasis, and immune escape (15, 16). Increasing evidence indicates that cytokine levels and their dynamic changes during PD-1 blockade are associated with treatment response, survival, and immune-related adverse events in patients with NSCLC (17). Nevertheless, the predictive significance of individual cytokines remains inconsistent across studies. This review summarizes the biological functions of major circulating cytokines, critically evaluates their value as dynamic biomarkers for predicting the efficacy and prognosis of PD-1 inhibitor therapy in NSCLC, and discusses current challenges and future directions for their clinical application.

## 2 TNF-α

Tumor necrosis factor-alpha (TNF-α), predominantly secreted by macrophages and T lymphocytes, is a pleiotropic cytokine that plays a pivotal yet paradoxical role in tumor immunity (18). Through binding to its distinct receptors, TNFR1 and TNFR2, TNF-α activates downstream signaling cascades, including the non-canonical nuclear factor-κB (NF-κB) pathway, thereby orchestrating tumor initiation, progression, and immune modulation (19, 20). The biological impact of TNF-α is highly context-dependent, exhibiting a “double-edged sword” effect. On the one hand, acute TNF-α signaling can promote inflammatory immune activation, facilitate cytotoxic T-cell recruitment, and induce tumor cell apoptosis (21, 22). On the other hand, chronic TNF-α exposure, particularly via TNFR2-mediated signaling, fosters an immunosuppressive microenvironment by expanding regulatory T (Treg) and regulatory B (Breg) cells, recruiting myeloid-derived suppressor cells, and ultimately facilitating tumor immune escape (23, 24). Given its profound immunomodulatory effects, circulating TNF-α has been extensively investigated as a predictive biomarker for PD-1 blockade in NSCLC, albeit with inconsistent findings (25). NSCLC patients with lower pre-treatment TNF-α levels may derive greater clinical benefits from PD-1 inhibitors (26). Conversely, when evaluating dynamic changes, Boutsikou et al. (27) reported that an increase in TNF-α levels following PD-1 blockade was associated with enhanced treatment responses and prolonged survival. However, Grecea et al. (28) observed no statistically significant correlation between TNF-α fluctuations and therapeutic outcomes. These discrepancies underscore the complexity of TNF-α biology and highlight that its predictive value may heavily depend on the timing of assessment and the prevailing immune context. Mechanistically, while TNF-α does not directly regulate PD-1/PD-L1 binding, it indirectly modulates the efficacy of PD-1 blockade through TNFR-mediated signaling (29, 30). The convergence of these clinical observations suggests that a controlled, transient elevation of TNF-α post-treatment—reflecting restored anti-tumor immune activation rather than chronic immunosuppression—may be beneficial (31, 32). Therefore, rather than serving as a static prognostic marker, the dynamic trajectory of serum TNF-α, which captures the delicate balance between immune reinvigoration and inflammatory toxicity, holds significant potential as a real-time indicator for monitoring PD-1 inhibitor efficacy and guiding personalized immunotherapy in NSCLC (24).

## 3 IFN-γ

**Figure 1:**
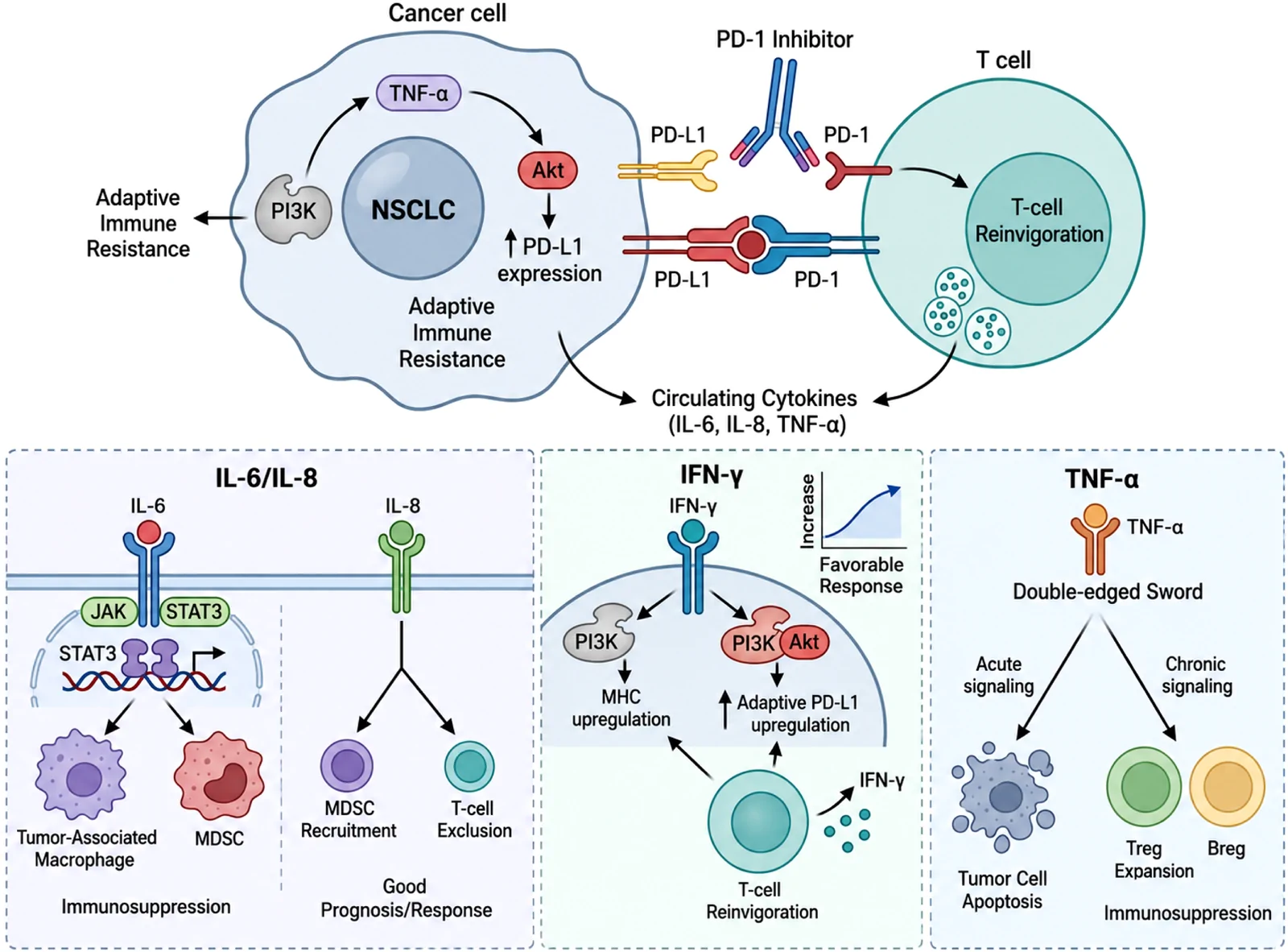
Circulating cytokines as potential dynamic biomarkers and prognosis in non-small cell lung cancer. PD-1 inhibitors disrupt the interaction between tumor-cell PD-L1 and T-cell PD-1, thereby restoring exhausted T-cell activity. However, cytokine-driven signaling can simultaneously promote antitumor immunity or induce adaptive immune resistance. In NSCLC cells, TNF-α-related PI3K/Akt signaling may increase PD-L1 expression and contribute to immune escape, whereas reinvigorated T cells release circulating cytokines that further shape treatment response. IL-6 activates the JAK/STAT3 pathway, promoting tumor-associated macrophages and myeloid-derived suppressor cells (MDSCs), while IL-8 facilitates MDSC recruitment and T-cell exclusion; persistent elevation of either cytokine is therefore generally associated with immunosuppression and an unfavorable response. IFN-γ enhances major histocompatibility complex expression and tumor antigen recognition, supporting T-cell reinvigoration and favorable responses, although sustained IFN-γ signaling may also activate PI3K/Akt-dependent adaptive PD-L1 upregulation. TNF-α exerts context-dependent, double-edged effects: acute signaling can induce tumor-cell apoptosis and enhance antitumor immunity, whereas chronic signaling promotes regulatory T-cell and regulatory B-cell expansion and reinforces immunosuppression. Collectively, the balance and dynamic changes of circulating IL-6, IL-8, IFN-γ, and TNF-α may reflect immune activation, adaptive resistance, and clinical response during PD-1 inhibitor therapy in NSCLC.

Interferon-gamma (IFN-γ), a type II interferon, exerts its immunoregulatory effects primarily via the Janus kinase/signal transducer and activator of transcription 3 (JAK/STAT3) and PI3K-Akt signaling pathways (33, 34). Upon PD-1 inhibition, the reinvigoration of exhausted cytotoxic T lymphocytes leads to IFN-γ secretion (35, 36). This cytokine subsequently amplifies tumor recognition by upregulating major histocompatibility complex (MHC) molecules and components of the antigen-processing machinery (37, 38). Genetic evidence further underscores its necessity; tumor models resistant to PD-1 inhibitors frequently harbor defects in the IFN-γ signaling pathway, and blocking IFN-γ receptors abrogates the antitumor efficacy of PD-1 blockade (39, 40). While IFN-γ inhibits tumor proliferation and angiogenesis, chronic or excessive IFN-γ signaling can drive adaptive immune resistance by transcriptionally upregulating PD-L1 on tumor and immune cells via JAK/STAT activation, thereby facilitating immune escape. Clinically, the predictive value of IFN-γ has been extensively evaluated, revealing a consensus on its prognostic value but significant divergence regarding its dynamic changes. IFN-γ expression is positively associated with patient prognosis, and that patients with high IFN-γ levels exhibit relatively longer progression-free survival (PFS) (41). Regarding on-treatment dynamics, however, findings remain conflicting. Kontakiotis et al. (42) observed that an increase in IFN-γ after PD-1 inhibitor treatment in patients with NSCLC was associated with improved overall survival. Conversely, Zhao et al. (43) have indicated the opposite trend, linking post-treatment IFN-γ elevation to disease progression in NSCLC and gastric cancer. Furthermore, certain studies have found no significant association between IFN-γ fluctuations and clinical benefit following anti-PD-1 therapy (44). Taken together, currently available evidence from both domestic and international studies suggests that elevated levels of IFN-γ and increased IFN-γ after treatment may be associated with clinical benefit from PD-1 inhibitors in patients with NSCLC. These discrepancies can be reconciled by considering the biological context and the magnitude of IFN-γ alteration (45, 46). The universal consensus is that intact IFN-γ signaling is an absolute prerequisite for effective PD-1 blockade. The divergence in dynamic studies likely reflects the difference between “moderate” and “excessive” immune activation (47, 48). A transient, moderate elevation of IFN-γ following PD-1 inhibition signifies successful T-cell reinvigoration and effective tumor clearance (49, 50). In contrast, persistent or excessive IFN-γ production may indicate the induction of adaptive immune resistance, chronic inflammation, and heightened immunological selection pressure, ultimately leading to treatment failure (51, 52). Therefore, rather than serving as a definitive static prognostic marker, the dynamic trajectory of serum TNF-α may have potential value as a real-time indicator for monitoring immune activation during PD-1 inhibitor therapy (**Figure 1**).

## 4 Interleukins

### 4.1 IL-6

IL-6 is secreted primarily by immune cells, including T helper 2 (Th2) cells and macrophages, as well as by endothelial cells, fibroblasts, and tumor cells (53, 54). Through activation of multiple signaling pathways, IL-6 participates in tumor-cell proliferation, differentiation, invasion, metastasis, and angiogenesis (55, 56). Mechanistically, IL-6 exerts its protumorigenic and immunosuppressive effects predominantly through the activation of the JAK/STAT3 signaling axis (57). In tumor cells, STAT3 activation drives proliferation, angiogenesis, and epithelial-mesenchymal transition (58, 59). Within the immune compartment, IL-6/STAT3 signaling impairs dendritic cell maturation and antigen presentation, while promoting the robust accumulation of myeloid-derived suppressor cells (MDSCs) and M2-polarized tumor-associated macrophages (60, 61). This cascade profoundly suppresses cytotoxic T lymphocyte (CTL) activity, thereby establishing a highly immunosuppressive milieu that inherently limits the reinvigoration of exhausted T cells following PD-1 blockade (62, 63). Serum IL-6 levels are elevated in patients with NSCLC compared with healthy individuals, suggesting that IL-6 may promote tumor proliferation, metastasis, and immune escape (64, 65). Crucially, the dynamic fluctuation of IL-6 during immunotherapy has emerged as a robust indicator of treatment efficacy. Clinical studies have consistently demonstrated that a post-treatment decline in serum IL-6 is strongly associated with improved progression-free survival (PFS) and overall survival (OS) in NSCLC patients receiving PD-1 inhibitors (66, 67). Therefore, a post-treatment decline in IL-6 following PD-1 inhibitor therapy may reflect reduced systemic inflammation, improved immune responsiveness, and partial restoration of antitumor T-cell function.

### 4.2 IL-8

Interleukin-8 (IL-8), a potent pro-inflammatory chemokine, is frequently overexpressed in various malignancies and plays a critical role in orchestrating an immunosuppressive tumor microenvironment (68). IL-8 exerts its protumorigenic effects primarily by recruiting MDSCs, tumor-associated macrophages (TAMs), and neutrophils into the TME (69, 70). This myeloid-enriched and T-cell-excluded phenotype impairs the infiltration and cytotoxic function of CD8^+^ T cells, thereby facilitating tumor immune evasion (71, 72). Furthermore, emerging evidence suggests that IL-8 may directly influence immune checkpoint pathways; for instance, IL-8 has been shown to upregulate PD-L1 expression via the STAT3/mTOR signaling cascade, highlighting the potential therapeutic rationale for targeting the IL-8 axis in combination with immunotherapy (73). Clinically, circulating IL-8 serves as a robust surrogate marker for both systemic inflammation and total tumor burden (74, 75). In advanced NSCLC, elevated serum IL-8 levels are consistently associated with aggressive disease progression and shortened overall survival (27, 76). More importantly, the dynamic trajectory of IL-8 during PD-1 inhibitor therapy has emerged as a compelling predictive biomarker. Studies have demonstrated that patients who maintain high serum IL-8 levels or exhibit a failure of IL-8 clearance following nivolumab treatment experience significantly poorer prognoses and primary or acquired resistance to PD-1 blockade (77–79). The convergence of these clinical observations can be mechanistically explained by the fundamental requirement of PD-1 inhibitors for pre-existing or inducible antitumor T-cell activity (80, 81). By fostering a T-cell-excluded TME and sustaining chronic systemic inflammation, elevated IL-8 creates a formidable barrier to T-cell reinvigoration, thereby blunting the therapeutic efficacy of PD-1 blockade (78, 82). While current evidence robustly links high on-treatment IL-8 to unfavorable outcomes, discrepancies among studies regarding optimal cutoff values, cancer types, and the precise timing of assessment still exist (78). Nevertheless, given its strong biological plausibility and clinical relevance, both baseline and dynamically monitored serum IL-8 hold substantial promise as predictive biomarkers for stratifying NSCLC patients undergoing PD-1 inhibitor therapy (17, 83), warranting further standardized investigation (**Supplementary Table 1**).

### 4.3 IL-17A

Interleukin-17A (IL-17A), predominantly secreted by T helper 17 (Th17) cells and highly enriched in the tumor microenvironment (TME), exerts its biological effects primarily through the activation of the JAK2/STAT3 and NF-κB signaling pathways (84). The role of IL-17A in cancer remains controversial. During early tumorigenesis, IL-17A appears to promote tumor growth, whereas in advanced malignant tissues, IL-17A may also enhance antitumor immune responses (85). This biological duality underscores the complexity of utilizing IL-17A as a predictive biomarker and necessitates a nuanced interpretation of its clinical significance. Serum IL-17A levels are higher in NSCLC patients than in healthy individuals (86). IL-17A is enriched in malignant squamous lung tumor tissue and is associated with poor clinical outcomes, possibly by upregulating PD-L1 expression and thereby impairing the efficacy of immunotherapy (87, 88). Zhao et al. (43) demonstrated that a post-treatment decline in IL-17A levels correlates strongly with clinical benefit in patients receiving PD-1 inhibitors. Furthermore, IL-17A is intricately linked to specific clinical comorbidities and toxicities. For instance, elevated serum IL-17A is frequently observed in NSCLC patients complicated by chronic obstructive pulmonary disease (COPD) and has been implicated in the pathogenesis of immune checkpoint inhibitor-related pneumonitis (89, 90). This highlights its potential not only in predicting therapeutic efficacy but also in monitoring immune-related adverse events (irAEs). From a mechanistic perspective, IL-17A predominantly influences PD-1 inhibitor efficacy by shaping a pro-inflammatory yet immunosuppressive TME (91, 92). By activating downstream pathways, it promotes tumor cell proliferation, angiogenesis, and the recruitment of neutrophils and other immunosuppressive cells, thereby driving inflammatory remodeling of the TME (93, 94). In lung cancer specifically, this IL-17A-mediated inflammatory milieu can compromise anti-tumor T-cell function, potentially by upregulating PD-L1 expression, which subsequently limits the reinvigoration of exhausted T cells by PD-1 blockade (87, 95).

### 4.4 Other interleukins

Beyond the extensively studied cytokines, the serum expression of other interleukins, including IL-1β, IL-2, IL-5, and IL-10, has also been implicated in the efficacy and prognosis of PD-1 inhibitor therapy in NSCLC (96). Rather than acting in isolation, these cytokines collectively orchestrate the delicate balance between immune activation and immunosuppression within the systemic and tumor microenvironments (97, 98). Evaluating their dynamic fluctuations provides complementary insights into the host’s immunological response to PD-1 blockade. IL-1β and IL-10 primarily contribute to an immunosuppressive and pro-tumorigenic milieu. High expression of IL-1β promotes chronic inflammation, angiogenesis, and the recruitment of immunosuppressive myeloid cells, thereby inducing T-cell dysfunction and indirectly reducing sensitivity to PD-1 inhibitors; consequently, elevated IL-1β is associated with poor prognosis in lung adenocarcinoma (67, 99). Similarly, IL-10 is a classic immunosuppressive cytokine that inhibits antigen presentation and effector T-cell activation. Zhao et al. (100) demonstrated that a decrease in serum IL-10 following PD-1 inhibitor treatment was significantly associated with therapeutic efficacy in NSCLC. This dynamic decline reflects the relief of systemic immunosuppression and the subsequent restoration of anti-tumor T-cell responses, highlighting the prognostic value of monitoring these inhibitory signals.

In contrast, IL-2 and IL-5 exhibit more complex, context-dependent roles. IL-2 is essential for T-cell proliferation and survival. While systemic IL-2 may inadvertently support regulatory T-cell (Treg) expansion depending on the dose and context, localized increases of IL-2 within the tumor microenvironment can synergistically enhance the expansion and cytotoxic activity of effector T cells. For instance, local IL-2 injection combined with systemic PD-1 blockade has shown synergistic anti-tumor effects in melanoma models (101). In the clinical setting of NSCLC, Costantini et al. (44) found that serum IL-2 levels were correlated with irAEs, suggesting its potential as a marker for immune hyperactivation, though its direct predictive value for efficacy requires further confirmation. Conversely, IL-5 is predominantly associated with Th2 immune responses and eosinophil biology. Zhao et al. (100) also reported that serum IL-5 levels were negatively correlated with PFS in NSCLC patients receiving PD-1 inhibitors, with responders exhibiting lower IL-5 levels at the time of best response. This negative association likely reflects a Th2-skewed immune polarization, which is generally unfavorable for the cytotoxic, Th1-driven anti-tumor immunity required for PD-1 inhibitor efficacy.

Serum expression of other cytokines, including IL-1β, IL-2, IL-5, and IL-10, has also been associated with the efficacy and prognosis of PD-1 inhibitor therapy in patients with NSCLC. For example, high IL-1β expression may be associated with poor prognosis in patients with lung adenocarcinoma. Langan et al. (101) reported a case in which local IL-2 injection combined with systemic PD-1 inhibitor therapy was used to treat malignant melanoma, suggesting that increased IL-2 within the tumor microenvironment may synergistically enhance the efficacy of PD-1 blockade. Costantini et al. (44) found that serum IL-2 levels were associated with immune checkpoint inhibitor-related adverse events, although the relationship between serum IL-2 and PD-1 inhibitor efficacy in NSCLC still requires further confirmation. Zhao et al. (100) showed that serum IL-5 levels were negatively correlated with PFS in patients with NSCLC treated with PD-1 inhibitors, and that in responders, IL-5 levels at the time of best response were lower than pre-treatment values. The same study also indicated that decreases in serum IL-10 after PD-1 inhibitor treatment were associated with treatment efficacy in NSCLC. These cytokines may also affect PD-1 blockade through distinct but interconnected mechanisms. IL-1β can promote chronic inflammation, angiogenesis, myeloid-cell recruitment, and T-cell dysfunction, thereby indirectly reducing sensitivity to PD-1 inhibitors (102–104). IL-2 is essential for T-cell proliferation and survival, and increased intratumoral IL-2 may enhance the expansion and cytotoxic activity of effector T cells, potentially synergizing with PD-1 blockade (105, 106). However, systemic IL-2 may also support regulatory T-cell expansion depending on dose and context (107, 108). IL-5 is mainly associated with type 2 immune responses and eosinophil biology, and its negative association with PFS may reflect a Th2-skewed immune state that is less favorable for cytotoxic antitumor immunity (109, 110). IL-10 is a classical immunosuppressive cytokine that inhibits antigen presentation and effector T-cell activation; therefore, decreased IL-10 after treatment may indicate relief of immunosuppression and improved response to PD-1 inhibitors (17, 111). These cytokines converge in reflecting the balance between immune activation and immune suppression, but their predictive value remains less established than that of IL-6 or IL-8 because available studies are relatively limited and often exploratory.

## 5 Conclusion

Serum cytokines represent a promising class of non-invasive biomarkers for evaluating the efficacy of PD-1 inhibitor therapy in NSCLC. Unlike tissue-based indicators, serum cytokines are readily accessible, amenable to repeated assessment, and capable of dynamically reflecting systemic immune activation, inflammatory burden, and tumor–host interactions during treatment. Current evidence suggests that decreases in IL-6, IL-8, IL-17A, and IL-10 after treatment may be associated with favorable therapeutic response, whereas high IL-8 and, in some settings, elevated IL-1β may indicate poor prognosis. By contrast, low TNF-α, increased TNF-α levels after treatment, and higher post-treatment IFN-γ levels may be associated with greater clinical benefit. These observations highlight the potential of serum cytokines to complement existing biomarkers and improve response assessment in patients undergoing PD-1 blockade.

Nevertheless, the clinical application of serum cytokines remains limited by substantial heterogeneity. This heterogeneity may arise from differences in NSCLC histological subtype, disease stage, smoking status, tumor burden, prior therapies, PD-L1 expression levels, treatment regimen, cytokine sampling time points, assay platforms, cutoff definitions, and systemic inflammatory or infectious conditions. In addition, several cytokines exhibit pleiotropic and even bidirectional effects in tumor biology, which complicates interpretation of their predictive significance. Future research should therefore prioritize large-scale prospective validation, standardized dynamic monitoring strategies, and integrative biomarker models that combine cytokine profiles with clinicopathological and molecular features. A deeper understanding of how serum cytokines reflect the evolving immune landscape during PD-1 inhibitor therapy may not only refine patient stratification, but also provide mechanistic insight into treatment response, resistance, and immune-related toxicity. Ultimately, serum cytokines may become valuable tools for advancing more precise and individualized immunotherapy in NSCLC.

## References

[1] Zhang Y Vaccarella S Morgan E Li M Etxeberria J Chokunonga E . Global variations in lung cancer incidence by histological subtype in 2020: a population-based study. Lancet Oncol. (2023) 24:1206–18. doi: 10.1016/s1470-2045(23)00444-8 37837979

[2] Roussos P Migkou M . Impact of PD-1/PD-L1 inhibitors on survival in stage III non-small-cell lung cancer: a systematic review. Cancer Pathogenesis Ther. (2024) 2:155–63. doi: 10.1016/j.cpt.2023.09.004 39027153 PMC11252514

[3] Ma S Nie H Wei C Jin C Wang L . Association between immune-related adverse events and prognosis in patients with advanced non-small cell lung cancer: a systematic review and meta-analysis. (2024) 14:1402017. doi: 10.3389/fonc.2024.1402017 PMC11109391 38779082

[4] Tang Q Chen Y Li X Long S Shi Y Yu Y . The role of PD-1/PD-L1 and application of immune-checkpoint inhibitors in human cancers. (2022) 13:964442. doi: 10.3389/fimmu.2022.964442 PMC9513184 36177034

[5] Lin X Kang K Chen P Zeng Z Li G Xiong W . Regulatory mechanisms of PD-1/PD-L1 in cancers. Mol Cancer. (2024) 23:108. doi: 10.1186/s12943-024-02023-w 38762484 PMC11102195

[6] Osawa T Yamada D Takao T Ming L Takarada T . PRRX1 upregulates PD-L1 in human mesenchymal stem cells. In Vitro Cell Dev Biol - Anim. (2024) 60:1132–7. doi: 10.1007/s11626-024-00911-5 38664281 PMC11655573

[7] Bai X Chen T Li Y Ge X Qiu C Gou H . PD-L1 expression levels in mesenchymal stromal cells predict their therapeutic values for autoimmune hepatitis. Stem Cell Res Ther. (2023) 14:370. doi: 10.1186/s13287-023-03594-z 38111045 PMC10729378

[8] Ettinger DS Wood DE Aisner DL Akerley W Bauman JR Bharat A . Non-small cell lung cancer, version 3.2022, NCCN clinical practice guidelines in oncology. J Natl Compr Canc Netw. (2022) 20:497–530. doi: 10.6004/jnccn.2022.0025 35545176

[9] Jiao J-M Liu C-G Zang D Chen J . Gut microbiota and metabolites: emerging prospects in the treatment of non-small cell lung cancer. (2025) 16:1638942. doi: 10.3389/fimmu.2025.1638942 PMC12627001 41268538

[10] Lee S-H Kim S Lee J Kim Y Joo Y Heo J . Comprehensive metabolomic analysis identifies key biomarkers and modulators of immunotherapy response in NSCLC patients. Drug Resistance Updates. (2024) 77:101159. doi: 10.1016/j.drup.2024.101159 39405736

[11] Yin X Zhang W Wang G Liu Y Dai B Yue L . The “double-edged sword” effect of cytokines in cancer: coexisting opportunities and challenges. (2025) 16:1701405. 10.3389/fimmu.2025.1701405 PMC12672462 41346580

[12] Marimuthu MMC Balamurugan BS Sundaram VA Anbalagan S Chopra H . Cytokine-based immunotherapy for gastric cancer: targeting inflammation for tumor control. Explor Target Antitumor Ther. (2025) 6:1002312. doi: 10.37349/etat.2025.1002312 40309351 PMC12040674

[13] Essogmo FE Zhilenkova AV Tchawe YSN Owoicho AM Rusanov AS Boroda A . Cytokine profile in lung cancer patients: anti-tumor and oncogenic cytokines. (2023) 15:5383. doi: 10.20944/preprints202308.1647.v1 PMC10670546 38001643

[14] Grecea-Balaj AM Soritau O Brie I Perde-Schrepler M Virág P Todor N . Immune cell–cytokine interplay in NSCLC and melanoma: A pilot longitudinal study of dynamic biomarker interactions. (2025) 5:29. doi: 10.3390/immuno5030029

[15] Yang L Fan Z Wang Z Zhou D . Soluble cytokines and chemokines in NSCLC: drivers of immune evasion and angiogenesis. (2026) 17:1699065. doi: 10.3389/fimmu.2026.1699065 PMC13011355 41884817

[16] Gong X Wang X Jin J Gong Z . The novel functions of chemokines in lung cancer progression. (2025) 16:1607225. doi: 10.3389/fimmu.2025.1607225 PMC12213639 40607416

[17] Kauffmann-Guerrero D Kahnert K Kiefl R Sellmer L Walter J Behr J . Systemic inflammation and pro-inflammatory cytokine profile predict response to checkpoint inhibitor treatment in NSCLC: a prospective study. Sci Rep. (2021) 11:10919. doi: 10.1038/s41598-021-90397-y 34035415 PMC8149421

[18] Jang DI Lee AH Shin HY Song HR Park JH Kang TB . The role of tumor necrosis factor alpha (TNF-α) in autoimmune disease and current TNF-α Inhibitors in therapeutics. Int J Mol Sci. (2021) 22:2719. doi: 10.3390/ijms22052719 33800290 PMC7962638

[19] Rodriguez BN Huang H Chia JJ Hoffmann A . The noncanonical NFκB pathway: Regulatory mechanisms in health and disease. WIREs Mech Dis. (2024) 16:e1646. doi: 10.1002/wsbm.1646 38634218 PMC11486840

[20] Ben-Baruch A . Tumor necrosis factor α: taking a personalized road in cancer therapy. (2022) 13:903679. doi: 10.3389/fimmu.2022.903679 PMC9157545 35663982

[21] van Loo G Bertrand MJM . Death by TNF: a road to inflammation. Nat Rev Immunol. (2023) 23:289–303. doi: 10.1038/s41577-022-00792-3 36380021 PMC9665039

[22] Chun N Ang RL Chan M Fairchild RL Baldwin WM Horwitz JK . T cell-derived tumor necrosis factor induces cytotoxicity by activating RIPK1-dependent target cell death. JCI Insight. (2021) 6:e148643. doi: 10.1172/jci.insight.148643 34752416 PMC8783689

[23] Okuzono Y Muraki Y Sato S . TNFR2 pathways are fully active in cancer regulatory T cells. Biosci Biotechnol Biochem. (2022) 86:351–61. doi: 10.1093/bbb/zbab226 35015831

[24] Medler J Kucka K Wajant H . Tumor necrosis factor receptor 2 (TNFR2): an emerging target in cancer therapy. Cancers (Basel). (2022) 14:2603. doi: 10.3390/cancers14112603 35681583 PMC9179537

[25] Benoot T Piccioni E De Ridder K Goyvaerts C . TNFα and immune checkpoint inhibition: friend or foe for lung cancer? Int J Mol Sci. (2021) 22:8691. doi: 10.3390/ijms22168691 34445397 PMC8395431

[26] Oyanagi J Koh Y Sato K Mori K Teraoka S Akamatsu H . Predictive value of serum protein levels in patients with advanced non-small cell lung cancer treated with nivolumab. Lung Cancer. (2019) 132:107–13. doi: 10.1016/j.lungcan.2019.03.020 31097082

[27] Belluomini L Cesta Incani U Smimmo A Avancini A Sposito M Insolda J . Prognostic impact of Interleukin-8 levels in lung cancer: A meta-analysis and a bioinformatic validation. Lung Cancer. (2024) 194:107893. doi: 10.1016/j.lungcan.2024.107893 39008934

[28] Grecea-Balaj AM Soritau O Brie I Perde-Schrepler M Virag P Fischer-Fodor E . Serum TNF -α, IL-10 and IL-2 trajectories and outcomes in NSCLC and melanoma under anti-PD-1 therapy: longitudinal real-world evidence from a single center. (2025) 47:746. doi: 10.3390/cimb47090746 PMC12468888 41020868

[29] Bertrand F Montfort A Marcheteau E Imbert C Gilhodes J Filleron T . TNFα blockade overcomes resistance to anti-PD-1 in experimental melanoma. Nat Commun. (2017) 8:2256. doi: 10.1038/s41467-017-02358-7 29273790 PMC5741628

[30] Yi M Li T Niu M Zhang H Wu Y Wu K . Targeting cytokine and chemokine signaling pathways for cancer therapy. Signal Transduction Targeted Ther. (2024) 9:176. doi: 10.1038/s41392-024-01868-3 39034318 PMC11275440

[31] Mercogliano MF Bruni S Mauro F Elizalde PV Schillaci R . Harnessing tumor necrosis factor alpha to achieve effective cancer immunotherapy. Cancers (Basel). (2021) 13:564. doi: 10.3390/cancers13030564 33540543 PMC7985780

[32] Virazels M Lusque A Brayer S Genais M Dufau C Milhès J . TNF signature in advanced melanoma patients treated with immune checkpoint inhibitors: Results from the MELANFα clinical study. Int J Cancer. (2025) 157:534–48. doi: 10.1002/ijc.35416 40098565 PMC12141980

[33] Wen C Lan Q Wang Y Ni Y Wong AST Liu D . Interferon signaling pathways in health and disease. Mol BioMed. (2025) 6:135. doi: 10.1186/s43556-025-00381-5 41359111 PMC12686292

[34] Tan YL Al-Masawa ME Eng SP Shafiee MN Law JX Ng MH . Therapeutic efficacy of interferon-gamma and hypoxia-primed mesenchymal stromal cells and their extracellular vesicles: underlying mechanisms and potentials in clinical translation. (2024) 12:1369. doi: 10.3390/biomedicines12061369 PMC11201753 38927577

[35] Nagasaki J Inozume T Sax N Ariyasu R Ishikawa M Yamashita K . PD-1 blockade therapy promotes infiltration of tumor-attacking exhausted T cell clonotypes. Cell Rep. (2022) 38:110331. doi: 10.1016/j.celrep.2022.110331 35108529

[36] Viramontes KM Neubert EN DeRogatis JM Tinoco R . PD-1 immune checkpoint blockade and PSGL-1 inhibition synergize to reinvigorate exhausted T cells. (2022) 13:869768. doi: 10.3389/fimmu.2022.869768 PMC9237324 35774790

[37] Cui J Zhang Y Yang L . IFN-gamma in the tumor microenvironment: dual roles in cancer progression and therapy. Excli J. (2025) 24:1352–71. doi: 10.17179/excli2025-8617 PMC12598110 41220920

[38] Chen Y Niu N Xue J . Double-edged roles of IFNγ in tumor elimination and immune escape. (2023) 06:8–17. doi: 10.1097/jp9.0000000000000113

[39] Shen H Huang F Zhang X Ojo OA Li Y Trummell HQ . Selective suppression of melanoma lacking IFN-γ pathway by JAK inhibition depends on T cells and host TNF signaling. Nat Commun. (2022) 13:5013. doi: 10.1038/s41467-022-32754-7 36008408 PMC9411168

[40] Wawrzyniak P Hartman ML . Dual role of interferon-gamma in the response of melanoma patients to immunotherapy with immune checkpoint inhibitors. Mol Cancer. (2025) 24:89. doi: 10.1186/s12943-025-02294-x 40108693 PMC11924818

[41] Kanai T Suzuki H Yoshida H Matsushita A Kawasumi H Samejima Y . Significance of quantitative interferon-gamma levels in non-small-cell lung cancer patients' Response to immune checkpoint inhibitors. Anticancer Res. (2020) 40:2787–93. doi: 10.21873/anticanres.14251 32366425

[42] Boutsikou E Domvri K Hardavella G Tsiouda D Zarogoulidis K Kontakiotis T . Tumour necrosis factor, interferon-gamma and interleukins as predictive markers of antiprogrammed cell-death protein-1 treatment in advanced non-small cell lung cancer: a pragmatic approach in clinical practice. Ther Adv Med Oncol. (2018) 10:1758835918768238. doi: 10.1177/1758835918768238 29662549 PMC5894896

[43] Zhao N Yi Y Cao W Fu X Mei N Li C . Serum cytokine levels for predicting immune-related adverse events and the clinical response in lung cancer treated with immunotherapy. Front Oncol. (2022) 12:923531. doi: 10.3389/fonc.2022.923531 36091125 PMC9449836

[44] Costantini A Julie C Dumenil C Hélias-Rodzewicz Z Tisserand J Dumoulin J . Predictive role of plasmatic biomarkers in advanced non-small cell lung cancer treated by nivolumab. Oncoimmunology. (2018) 7:e1452581. doi: 10.1080/2162402x.2018.1452581 30221046 PMC6136870

[45] Ding H Wang G Yu Z Sun H Wang L . Role of interferon-gamma (IFN-γ) and IFN-γ receptor 1/2 (IFNγR1/2) in regulation of immunity, infection, and cancer development: IFN-γ-dependent or independent pathway. Biomedicine Pharmacotherapy. (2022) 155:113683. doi: 10.1016/j.biopha.2022.113683 36095965

[46] Han J Wu M Liu Z . Dysregulation in IFN-γ signaling and response: the barricade to tumor immunotherapy. (2023) 14:1190333. doi: 10.3389/fimmu.2023.1190333 PMC10233742 37275859

[47] Holzgruber J Martins C Kulcsar Z Duplaine A Rasbach E Migayron L . Type I interferon signaling induces melanoma cell-intrinsic PD-1 and its inhibition antagonizes immune checkpoint blockade. Nat Commun. (2024) 15:7165. doi: 10.1038/s41467-024-51496-2 39187481 PMC11347607

[48] Karri V Dalia SM . Persistent IFN-γ signaling in acquired resistance to PD-(L)1 blockade in NSCLC. (2025) 9:352–8. doi: 10.20517/jtgg.2025.90

[49] Wong CW Huang YY Hurlstone A . The role of IFN-γ-signalling in response to immune checkpoint blockade therapy. Essays Biochem. (2023) 67:991–1002. doi: 10.1042/ebc20230001 37503572 PMC10539948

[50] Anagnostou V Forde PM White JR Niknafs N Hruban C Naidoo J . Dynamics of tumor and immune responses during immune checkpoint blockade in non-small cell lung cancer. Cancer Res. (2019) 79:1214–25. doi: 10.1158/0008-5472.can-18-1127 30541742 PMC6432636

[51] Memon D Schoenfeld AJ Ye D Fromm G Rizvi H Zhang X . Clinical and molecular features of acquired resistance to immunotherapy in non-small cell lung cancer. Cancer Cell. (2024) 42:209–224.e209. doi: 10.1136/jitc-2022-sitc2022.0522 PMC11249385 38215748

[52] Chai R Yin Y Cai X Fu X Zhang Q . Patterns of failure in patients with advanced non-small cell lung cancer treated with immune checkpoint inhibitors. Front Oncol. (2021) 11:724722. doi: 10.3389/fonc.2021.724722 34557412 PMC8454403

[53] Grebenciucova E VanHaerents S . Interleukin 6: at the interface of human health and disease. (2023) 14:1255533. doi: 10.3389/fimmu.2023.1255533 PMC10569068 37841263

[54] Li Y Zhao J Yin Y Li K Zhang C Zheng Y . The role of IL-6 in fibrotic diseases: molecular and cellular mechanisms. Int J Biol Sci. (2022) 18:5405–14. doi: 10.7150/ijbs.75876 36147459 PMC9461670

[55] Zhou Y Song X Yuan M Li Y . The diverse function of IL-6 in biological processes and the advancement of cancer. Immune Netw. (2025) 25:e22. doi: 10.4110/in.2025.25.e22 40620420 PMC12226255

[56] Rašková M Lacina L Kejík Z Venhauerová A Skaličková M Kolář M . The role of IL-6 in cancer cell invasiveness and metastasis-overview and therapeutic opportunities. Cells. (2022) 11:3698. doi: 10.3390/cells11223698 PMC9688109 36429126

[57] Liang Y Shao Y Gu W . Role of interleukin-6 in resistance to tumor therapy. Discov Oncol. (2025) 16:1791. doi: 10.1007/s12672-025-03644-3 41026295 PMC12484520

[58] Parakh S Ernst M Poh AR . Multicellular effects of STAT3 in non-small cell lung cancer: mechanistic insights and therapeutic opportunities. Cancers (Basel). (2021) 13:6228. doi: 10.3390/cancers13246228 34944848 PMC8699548

[59] Zhao J Wang X Mi Z Jiang X Sun L Zheng B . STAT3/miR-135b/NF-κB axis confers aggressiveness and unfavorable prognosis in non-small-cell lung cancer. Cell Death Dis. (2021) 12:493. doi: 10.1038/s41419-021-03773-x 33990540 PMC8121828

[60] Papavassiliou KA Marinos G Papavassiliou AG . Combining STAT3-targeting agents with immune checkpoint inhibitors in NSCLC. Cancers (Basel). (2023) 15:386. doi: 10.3390/cancers15020386 36672335 PMC9857288

[61] Wang B Pan L Chen M Ma Y Gao J Tang D . SIRP-alpha-IL-6 axis induces immunosuppressive macrophages in non-small-cell lung cancer. Biochem Biophys Res Commun. (2023) 682:386–96. doi: 10.1016/j.bbrc.2023.10.035 37844448

[62] Kuo IY Yang YE Yang PS Tsai YJ Tzeng HT Cheng HC . Converged Rab37/IL-6 trafficking and STAT3/PD-1 transcription axes elicit an immunosuppressive lung tumor microenvironment. Theranostics. (2021) 11:7029–44. doi: 10.7150/thno.60040 34093869 PMC8171097

[63] Zhang L Guo X Sun X Liao J Liu Q Ye Y . Analysis of tumor-infiltrating exhausted T cells highlights IL-6 and PD1 blockade as a combined immunotherapy strategy for non-small cell lung cancer. (2025) 16:1486329. doi: 10.3389/fimmu.2025.1486329 PMC11876966 40040705

[64] Jasemi SV Zia S Mirbahari SG Sadeghi M . A systematic review and meta-analysis to evaluate blood levels of interleukin-6 in lung cancer patients. Kardiochir Torakochirurgia Pol. (2023) 20:240–50. doi: 10.5114/kitp.2023.134177 PMC10809806 38283553

[65] Wang Y Guo Z Xu A Fu Z Hou Y Tang K . Mechanistic insights into IL-6-mediated NK cell dysfunction in NSCLC via the IRE1α-XBP1s-STAT3-UBE2S axis. NPJ Precis Oncol. (2025) 9:361. doi: 10.1038/s41698-025-01140-z 41254082 PMC12627650

[66] Keegan A Ricciuti B Garden P Cohen L Nishihara R Adeni A . Plasma IL-6 changes correlate to PD-1 inhibitor responses in NSCLC. J Immunother Cancer. (2020) 8:678. doi: 10.1136/jitc-2020-000678 33020238 PMC7537334

[67] Shi Y Liu X Du J Zhang D Liu J Chen M . Circulating cytokines associated with clinical outcomes in advanced non-small cell lung cancer patients who received chemoimmunotherapy. Thorac Cancer. (2022) 13:219–27. doi: 10.1111/1759-7714.14248 34825500 PMC8758427

[68] Meier C Brieger A . The role of IL-8 in cancer development and its impact on immunotherapy resistance. Eur J Cancer. (2025) 218:115267. doi: 10.1016/j.ejca.2025.115267 39899909

[69] Qin R Ren W Ya G Wang B He J Ren S . Role of chemokines in the crosstalk between tumor and tumor-associated macrophages. Clin Exp Med. (2023) 23:1359–73. doi: 10.1007/s10238-022-00888-z 36173487 PMC10460746

[70] Gu X Zhu Y Su J Wang S Su X Ding X . Lactate-induced activation of tumor-associated fibroblasts and IL-8-mediated macrophage recruitment promote lung cancer progression. Redox Biol. (2024) 74:103209. doi: 10.1016/j.redox.2024.103209 38861833 PMC11215341

[71] Han ZJ Li YB Yang LX Cheng HJ Liu X Chen H . Roles of the CXCL8-CXCR1/2 axis in the tumor microenvironment and immunotherapy. Molecules. (2021) 27:137. doi: 10.3390/molecules27010137 35011369 PMC8746913

[72] Shao Y Lan Y Chai X Gao S Zheng J Huang R . CXCL8 induces M2 macrophage polarization and inhibits CD8(+) T cell infiltration to generate an immunosuppressive microenvironment in colorectal cancer. FASEB J. (2023) 37:e23173. doi: 10.1096/fj.202201982rrr 37665572

[73] Luo D Zhou J Ruan S Zhang B Zhu H Que Y . Overcoming immunotherapy resistance in gastric cancer: insights into mechanisms and emerging strategies. Cell Death Dis. (2025) 16:75. doi: 10.1038/s41419-025-07385-7 39915459 PMC11803115

[74] Scaglione IM Bazzichetto C Borghesani M Zacchi F Eccher S Sposito M . Interleukin-8: a tumor-agnostic biomarker integrating cancer biology and host response across solid tumors. Cancer Treat Rev. (2026) 146:103135. doi: 10.1016/j.ctrv.2026.103135 41997080

[75] Carril-Ajuria L Flippot R Naigeon M Dalban C Desnoyer A Rioux-Leclercq N . High circulating IL-6/IL-8 is associated with intratumoral myeloid contexture and poor outcomes in patients with advanced renal cell carcinoma treated with PD-1 blockade. Clin Cancer Res. (2025) 31:4150–8. doi: 10.1158/1078-0432.ccr-24-0902 40762801

[76] Favaro F Luciano-Mateo F Moreno-Caceres J Hernández-Madrigal M Both D Montironi C . TRAIL receptors promote constitutive and inducible IL-8 secretion in non-small cell lung carcinoma. Cell Death Dis. (2022) 13:1046. doi: 10.1038/s41419-022-05495-0 36522309 PMC9755151

[77] Agulló-Ortuño MT Gómez-Martín Ó Ponce S Iglesias L Ojeda L Ferrer I . Blood predictive biomarkers for patients with non-small-cell lung cancer associated with clinical response to nivolumab. Clin Lung Cancer. (2020) 21:75–85. doi: 10.1016/j.cllc.2019.08.006 31562055

[78] Zou D Song A Yong W . Prognostic role of IL-8 in cancer patients treated with immune checkpoint inhibitors: a system review and meta-analysis. (2023) 13:1176574. doi: 10.3389/fonc.2023.1176574 PMC10446970 37621675

[79] Liu H Zhou C Jiang H Chu T Zhong R Zhang X . Prognostic role of serum cytokines level in non-small cell lung cancer patients with anti-PD-1 and chemotherapy combined treatment. (2024) 15:1430301. doi: 10.3389/fimmu.2024.1430301 PMC11534701 39502692

[80] Ribas A . Basic rules to respond to PD-1 blockade cancer immunotherapy. J Immunother Cancer. (2025) 13:e012096. doi: 10.1136/jitc-2025-012096 40425230 PMC12107557

[81] Li H van der Merwe PA Sivakumar S . Biomarkers of response to PD-1 pathway blockade. Br J Cancer. (2022) 126:1663–75. doi: 10.1038/s41416-022-01743-4 35228677 PMC9174485

[82] Zahid KR Raza U Tumbath S Jiang L Xu W Huang X . Neutrophils: musketeers against immunotherapy. (2022) 12:975981. doi: 10.3389/fonc.2022.975981 PMC9453237 36091114

[83] Schalper KA Carleton M Zhou M Chen T Feng Y Huang S-P . Elevated serum interleukin-8 is associated with enhanced intratumor neutrophils and reduced clinical benefit of immune-checkpoint inhibitors. Nat Med. (2020) 26:688–92. doi: 10.1038/s41591-020-0856-x 32405062 PMC8127102

[84] Shibabaw T Teferi B Ayelign B . The role of Th-17 cells and IL-17 in the metastatic spread of breast cancer: as a means of prognosis and therapeutic target. (2023) 14:1094823. doi: 10.3389/fimmu.2023.1094823 PMC10040566 36993955

[85] Vitiello GA Miller G . Targeting the interleukin-17 immune axis for cancer immunotherapy. J Exp Med. (2020) 217:e20190456. doi: 10.1084/jem.20190456 31727783 PMC7037254

[86] Chen G Zhang PG Li JS Duan JJ Su W Guo SP . Th17 cell frequency and IL-17A production in peripheral blood of patients with non-small-cell lung cancer. J Int Med Res. (2020) 48:300060520925948. doi: 10.1177/0300060520925948 32600079 PMC7328058

[87] Liao H Chang X Gao L Ye C Qiao Y Xie L . IL-17A promotes tumorigenesis and upregulates PD-L1 expression in non-small cell lung cancer. J Transl Med. (2023) 21:828. doi: 10.1186/s12967-023-04365-3 37978543 PMC10656985

[88] Xu R Ke X Shang W Liu S Fu X Wang T . Distribution and clinical significance of IL-17A in tumor-infiltrating lymphocytes of non-small cell lung cancer patients. Pathol Oncol Res. (2022) 28:1610384. doi: 10.3389/pore.2022.1610384 35665407 PMC9156623

[89] Wang X She X Gao W Liu X Shi B . Correlation between the Treg/Thl7 index and the efficacy of PD-1 monoclonal antibody in patients with advanced non-small-cell lung cancer complicated with chronic obstructive pulmonary disease. Comput Math Methods Med. (2022) 2022:2923998. doi: 10.1155/2022/2923998 35915772 PMC9338744

[90] Wang YN Lou DF Li DY Jiang W Dong JY Gao W . Elevated levels of IL-17A and IL-35 in plasma and bronchoalveolar lavage fluid are associated with checkpoint inhibitor pneumonitis in patients with non-small cell lung cancer. Oncol Lett. (2020) 20:611–22. doi: 10.3892/ol.2020.11618 32565986 PMC7285943

[91] Liu C Liu R Wang B Lian J Yao Y Sun H . Blocking IL-17A enhances tumor response to anti-PD-1 immunotherapy in microsatellite stable colorectal cancer. J Immunother Cancer. (2021) 9:1895. doi: 10.1136/jitc-2020-001895 33462141 PMC7813395

[92] Li S Na R Li X Zhang Y Zheng T . Targeting interleukin-17 enhances tumor response to immune checkpoint inhibitors in colorectal cancer. Biochim Biophys Acta (BBA) - Rev Cancer. (2022) 1877:188758. doi: 10.1016/j.bbcan.2022.188758 35809762

[93] Khalid F Takagi K Sato A Yamaguchi M Guestini F Miki Y . Interleukin (IL)-17A in triple-negative breast cancer: a potent prognostic factor associated with intratumoral neutrophil infiltration. Breast Cancer. (2023) 30:748–57. doi: 10.1007/s12282-023-01467-0 37178415

[94] Akbay EA Koyama S Liu Y Dries R Bufe LE Silkes M . Interleukin-17A promotes lung tumor progression through neutrophil attraction to tumor sites and mediating resistance to PD-1 blockade. J Thorac Oncol. (2017) 12:1268–79. doi: 10.1016/j.jtho.2017.09.925 28483607 PMC5532066

[95] Li Q Ngo PT Egilmez NK . Anti-PD-1 antibody-mediated activation of type 17 T-cells undermines checkpoint blockade therapy. Cancer Immunol Immunother. (2021) 70:1789–96. doi: 10.1007/s00262-020-02795-2 33245376 PMC10991855

[96] Su S Chen F Lv X Qi L Ding Z Ren W . Predictive value of peripheral blood biomarkers in patients with non-small-cell lung cancer responding to anti-PD-1-based treatment. Cancer Immunol Immunother. (2024) 73:12. doi: 10.1007/s00262-023-03620-2 38231411 PMC10794255

[97] Trujillo-Cirilo L Weiss-Steider B Vargas-Angeles CA Corona-Ortega MT Rangel-Corona R . Immune microenvironment of cervical cancer and the role of IL-2 in tumor promotion. Cytokine. (2023) 170:156334. doi: 10.1016/j.cyto.2023.156334 37598478

[98] Kiss M Vande Walle L Saavedra PHV Lebegge E Van Damme H Murgaski A . IL1β promotes immune suppression in the tumor microenvironment independent of the inflammasome and gasdermin D. Cancer Immunol Res. (2021) 9:309–23. doi: 10.1158/2326-6066.cir-20-0431 33361087

[99] Ding X Zhang J Shi M Liu D Zhang L Zhang R . High expression level of interleukin-1β is correlated with poor prognosis and PD-1 expression in patients with lung adenocarcinoma. Clin Transl Oncol. (2021) 23:35–42. doi: 10.1007/s12094-020-02392-w 32472456

[100] Zhao Q Bi Y Sun H Xiao M . Serum IL-5 and IFN-γ are novel predictive biomarkers for anti-PD-1 treatment in NSCLC and GC patients. Dis Markers. (2021) 2021:5526885. doi: 10.1155/2021/5526885 34239620 PMC8235970

[101] Langan EA Kümpers C Graetz V Perner S Zillikens D Terheyden P . Intralesional interleukin-2: a novel option to maximize response to systemic immune checkpoint therapy in loco-regional metastatic melanoma. Dermatol Ther. (2019) 32:e12901. doi: 10.1111/dth.12901 30974014

[102] Diwanji R O'Brien NA Choi JE Nguyen B Laszewski T Grauel AL . Targeting the IL1β pathway for cancer immunotherapy remodels the tumor microenvironment and enhances antitumor immune responses. Cancer Immunol Res. (2023) 11:777–91. doi: 10.1158/2326-6066.cir-22-0290 37040466

[103] Aggen DH Ager CR Obradovic AZ Chowdhury N Ghasemzadeh A Mao W . Blocking IL1 beta promotes tumor regression and remodeling of the myeloid compartment in a renal cell carcinoma model: multidimensional analyses. Clin Cancer Res. (2021) 27:608–21. doi: 10.1158/1078-0432.ccr-20-1610 33148676 PMC7980495

[104] Hirayama A Tanaka K Tsutsumi H Nakanishi T Yamashita S Mizusaki S . Regulation of PD-L1 expression in non-small cell lung cancer by interleukin-1β. Front Immunol. (2023) 14:1192861. doi: 10.3389/fimmu.2023.1192861 37441079 PMC10333574

[105] Ren Z Zhang A Sun Z Liang Y Ye J Qiao J . Selective delivery of low-affinity IL-2 to PD-1+ T cells rejuvenates antitumor immunity with reduced toxicity. J Clin Invest. (2022) 132:e153604. doi: 10.1136/jitc-2022-sitc2022.1236 PMC8803347 35104810

[106] Kaptein P Slingerland N Metoikidou C Prinz F Brokamp S Machuca-Ostos M . CD8-targeted IL2 unleashes tumor-specific immunity in human cancer tissue by reviving the dysfunctional T-cell pool. Cancer Discov. (2024) 14:1226–51. doi: 10.1158/2159-8290.cd-23-1263 38563969 PMC11215409

[107] Harris F Berdugo YA Tree T . IL-2-based approaches to Treg enhancement. Clin Exp Immunol. (2023) 211:149–63. doi: 10.1093/cei/uxac105 36399073 PMC10019135

[108] Wang J Zhang S-X Chang J-S Cheng T Jiang X-J Su Q-Y . Low-dose IL-2 improved clinical symptoms by restoring reduced regulatory T cells in patients with refractory rheumatoid arthritis: a randomized controlled trial. (2022) 13:947341. doi: 10.3389/fimmu.2022.947341 PMC9744779 36524114

[109] Sibille A Corhay JL Louis R Ninane V Jerusalem G Duysinx B . Eosinophils and lung cancer: from bench to bedside. Int J Mol Sci. (2022) 23:5066. doi: 10.3390/ijms23095066 35563461 PMC9101877

[110] Frafjord A Buer L Hammarström C Aamodt H Woldbæk PR Brustugun OT . The immune landscape of human primary lung tumors is Th2 skewed. (2021) 12:764596. doi: 10.3389/fimmu.2021.764596 PMC8637168 34868011

[111] Sun Z Fourcade J Pagliano O Chauvin JM Sander C Kirkwood JM . IL10 and PD-1 cooperate to limit the activity of tumor-specific CD8+ T cells. Cancer Res. (2015) 75:1635–44. doi: 10.1158/0008-5472.can-14-3016 25720800 PMC4401638

